# Perinatal Cannabis Use, Depression, and the Mother-Child Dyad: Protocol for a Prospective Multimethod Study

**DOI:** 10.2196/71302

**Published:** 2025-12-11

**Authors:** Lauren Micalizzi, Lindy K Howe, Cynthia L Battle, Jane Metrik, Rachel L Gunn

**Affiliations:** 1 Center for Alcohol and Addiction Studies Department of Behavioral and Social Sciences Brown University Providence, RI United States; 2 Department of Psychiatry and Human Behavior Brown University Providence, RI United States

**Keywords:** postpartum depression, PPD, perinatal cannabis use, mother-infant relationship, ecological momentary assessment, EMA, maternal mental health

## Abstract

**Background:**

Postpartum depression (PPD) rates in the United States are among the highest globally, and PPD can pose significant, long-term risks to families. Concurrently, perinatal cannabis use is increasing in prevalence and may exacerbate PPD. Although evidence links cannabis use with PPD, little is known about its impact on immediate depressive symptoms or depression trajectories across the perinatal period. Moreover, the potential impact of cannabis use on mother-child attachment, bonding, and emotional availability could intensify the effects of cannabis on PPD.

**Objective:**

This protocol study is a longitudinal investigation aimed at detecting initial signals of the daily and long-term associations between cannabis use, PPD symptoms, and the mother-infant relationship.

**Methods:**

Participants (N=20) were individuals carrying a singleton pregnancy who reported using cannabis at least twice weekly. Recruitment was through community outreach and online advertisements. Study participation began with a baseline laboratory assessment during pregnancy, which included surveys on mental health and substance use. Follow-ups were conducted virtually at 6 weeks post partum and in the laboratory at 6 months post partum and included additional surveys on infant development, aspects of the mother-infant relationship (eg, attachment), as well as behavioral interaction tasks. Each assessment was paired with a 2-week ecological momentary assessment burst, resulting in three bursts. To support retention, brief check-in visits were completed during the second and third trimesters (depending on gestational age at enrollment), and a postdelivery phone call was conducted within 2 weeks of delivery. A 2-level linear mixed-effect models will be used to examine both event-level and person-level associations of cannabis use with momentary negative affect, PPD symptoms, and attachment, bonding, and emotional availability. Interaction models will test whether these characteristics of the mother-child relationship intensify the association between cannabis use and PPD symptoms.

**Results:**

This project received institutional review board approval on December 19, 2022, and was awarded funding on February 1, 2023. The recruitment goal of 20 participants was reached on September 4, 2024. Recruitment challenges were encountered early in the study, leading to successful adaptations in recruitment and data collection protocols. Follow-up data collection is ongoing, with completion expected by October 2025 and results anticipated by April 2026. Retention rates approach 100% at follow-up, and ecological momentary assessment compliance rates exceed those observed in nonpregnant samples (ie, >80%).

**Conclusions:**

This protocol study demonstrates our ability to collect momentary and longitudinal data to examine the daily and cumulative impact of cannabis use on PPD and the mother-infant relationship. These data are well-positioned to provide preliminary evidence on how cannabis use may shape depressive symptoms during a particularly high-risk period for maternal mental health. The findings will inform a larger-scale study and advance understanding of the potential effects of cannabis use on perinatal mental health.

**International Registered Report Identifier (IRRID):**

DERR1-10.2196/71302

## Introduction

### Background

Rates of postpartum depression (PPD) in the United States are among the highest in the world [1-3]. It is estimated that one in 7 birthing individuals experiences perinatal depression (depressive episodes that occur during pregnancy or the first postpartum year) which can pose profound, protracted risks to the mother, child, and family [4,5]. PPD is the leading complication related to childbearing, and suicide rates in the postnatal period may be as high as other times in a woman’s life [6,7]. Further, PPD is a chronic, relapsing disease; those who experience PPD are 300 times more at risk of experiencing PPD in subsequent pregnancies and twice as likely to experience depression within 5 years post partum relative to those who do not experience PPD in the first 12 months after birth [8]. An additional consequence of PPD may be disruption to the mother-child relationship, which can result in a developmental cascade of unhealthy outcomes for the child [9].

### Perinatal Cannabis Use

Perinatal cannabis use is increasing in prevalence [10] and may worsen PPD over time. Relative to pregnant people without depression, pregnant people with depression are at least 3 times as likely to use cannabis during pregnancy and at least 2 times as likely to use cannabis while breastfeeding [11]. Pregnant people report that cannabis use helps them cope with depressive symptoms [12]. While cannabis use may reduce depressive symptoms, such as negative affect in the moment [2], it may increase these symptoms over time, as has been demonstrated in nonpregnant samples [13]. Also, in nonpregnant samples, using cannabis to cope is linked to cannabis dependence and increased depression [14]. There is initial evidence that those who engage in prenatal cannabis use experience higher levels of PPD and anxiety [15,16], but the impact of prenatal cannabis use on longitudinal patterns of PPD have not been assessed, which is essential to mitigate PPD and inform treatment. Examination of longitudinal trajectories are important, as PPD persists at 6 months post partum in 50% of mothers [17].

Although this directional association (cannabis use leading to PPD) is theorized and is the focus of this study, a bidirectional relationship may exist. As such, research is needed on both the momentary and long-term effects of cannabis use on PPD. Intensive longitudinal designs that capture momentary associations as well as long-term changes in symptoms can offer a rigorous approach to inform hypothesized directionality and momentary impact of cannabis use on PPD symptoms.

### Postpartum Cannabis Use, Depression, and the Mother-Child Relationship

Maternal well-being is essential for attentive, responsive, and emotionally available parenting [18], and both substance use and depression can interfere with a mother’s ability to provide this level of care. The relationship between a mother and their offspring begins before birth, with maternal-fetal attachment reflecting a pregnant person’s emotional bond and cognitive representations of the developing fetus (eg, imagining interactions or future scenarios). This attachment often translates into behaviors that convey care for the fetus, including nurturance (eg, abstaining from harmful substances), comforting (eg, touching or holding the belly), and preparing physically and emotionally for the baby’s arrival [19]. Evidence indicates that maternal-fetal attachment is strongly associated with both depressed mood and substance use during pregnancy [19,20], but the literature on cannabis use and maternal-fetal attachment is lacking.

After delivery, early relational processes evolve into measurable aspects of the postnatal mother-infant relationship, including bonding and emotional availability. Bonding refers to a mother’s emotional connectedness toward their child, and is evidenced by the provision of protection, comfort, and care [21]. Emotional availability is a dyadic construct capturing how well caregiver and child can respond to each other’s emotional cues in ways that foster a healthy relationship [22]. A dyadic system that is not functioning well may present with a restricted range of emotion or lack of interest or sustained pleasure [22]. Thus, bonding reflects the caregiver’s inner emotional experience, and emotional availability may reflect the manifestation of these feelings or the quality of the observed relationship in action. These constructs are central to healthy relational development and may be particularly vulnerable to disruption in the context of maternal cannabis use and PPD.

There is overlap in the neurobiological and psychological systems that are implicated in substance use and mother-infant interactions (eg, emotional responsivity, reward processing, and executive functioning [23]). Consequently, substance use can lower intensity of maternal feelings toward the infant, which can result in less responsivity to infant cues [24]. Systematic reviews and meta-analyses [24-28] consistently demonstrate the significant negative consequences of PPD on various aspects of the mother-infant relationship. Depression-related anhedonia and low or dysregulated affect can interfere with a mother’s attunement to their infant and disrupt both bonding [29] and emotional availability [30]. For example, there is evidence that depressed (vs nondepressed) mothers engage in less communication and smiling toward their infants [31], as well as less physical touch during social interactions [32]. There are likely bidirectional associations between depression and mother-infant interaction quality that can be probed with momentary data. None of this work, to our knowledge, has focused on prenatal cannabis use, specifically. Given what is established for the use of other substances, cannabis use may operate similarly, via mechanisms like affect blunting, disrupting emotional regulation and sensitivity, increasing withdrawal or distraction, or via neurobiological impacts of tetrahydrocannabinol on mood and cognition. Extending this work to cannabis use is a critical next step, as the rapidly evolving status of cannabis legalization is tied to increased access and decreased perceived harm, both of which predict increased use among pregnant people [33]. Moreover, research has yet to examine how cannabis use affects the developing relationship between mother and child, and how the interactions between the 2 influence PPD. There is the strong potential for a cyclical relationship, such that disrupted interactions may perpetuate a negative feedback loop such that PPD leads to negative mother-infant interactions, which exacerbates PPD. Research is needed to test this hypothesized effect as well as factors that may exacerbate PPD.

Another opportunity lies in multimethod approaches (ie, behavioral, self-report, and ecological momentary assessment [EMA]) that yield the most comprehensive data on maternal-child interaction quality, as maternal perceptions and observational assessments may capture different aspects of the mother-infant relationship [34]. Cannabis use is common in the postpartum period, but controlled cannabis administration cannot be studied due to ethical concerns. The use of EMA is therefore feasible as it facilitates the understanding of the impact of cannabis on mother-child interactions as they naturally occur.

### Use of Ecologically Valid Methods for Studying Perinatal Cannabis Use

EMA methods are ecologically valid and ideal for studying cannabis use, PPD, and mother-child interactions during the perinatal period, as they limit recall bias and allow for low-burden assessment within an individual’s typical context [35,36]. Further, EMA methods are essential for examining the impact of cannabis use in a pregnant sample for whom administration of substances is not possible, but allows for the harnessing of the frequent cannabis use already occurring in this sample. Finally, these intensive longitudinal data collection methods allow for the examination of within- and between-person sources of variation that exist for cannabis use across the perinatal period. While cross-sectional research has been beneficial in unearthing associations among cannabis use, maternal health outcomes, and mother-infant interactions, there remains a gap in our understanding of these naturalistic relationships. EMA capture of parenting is essential to bolster laboratory and self-assessment and capture mother-infant interactions as they occur in the real world; yet no studies have used EMA methods to examine how cannabis use and PPD impact the mother-child relationship. This study answers a call for the use of ecologically valid methods to study caregiver-child interactions in the natural environment [37].

### Current Study

Despite mounting evidence that cannabis use cooccurs with PPD, we have very little understanding of the impact of cannabis use on the immediate experience of PPD symptoms (such as negative affect) or trajectories across the perinatal period. Further, the influence of cannabis use on attachment, bonding, and emotional availability in the mother-child relationship may be profound and may exacerbate the effect of cannabis use on PPD. The first objective of this study is to gather pilot data to examine initial direction of effects for the event-level and longitudinal impact of cannabis use on PPD symptoms. It is hypothesized that (1) cannabis use will result in decreased negative affect in the moment, and (2) more frequent cannabis use will increase depression symptoms across the postnatal period. The second objective is to evaluate the impact of cannabis use on mother-infant relationship and PPD via multiple methods, and it is hypothesized that (3) cannabis use will be associated with less bonding and lower emotional availability at the day-level and across the perinatal period (assessed both in the natural environment and laboratory), and (4) lower-quality mother-child relationships are expected to exacerbate the association between maternal cannabis use and PPD across the postpartum period.

## Methods

### Design Overview

**Figure 1:**
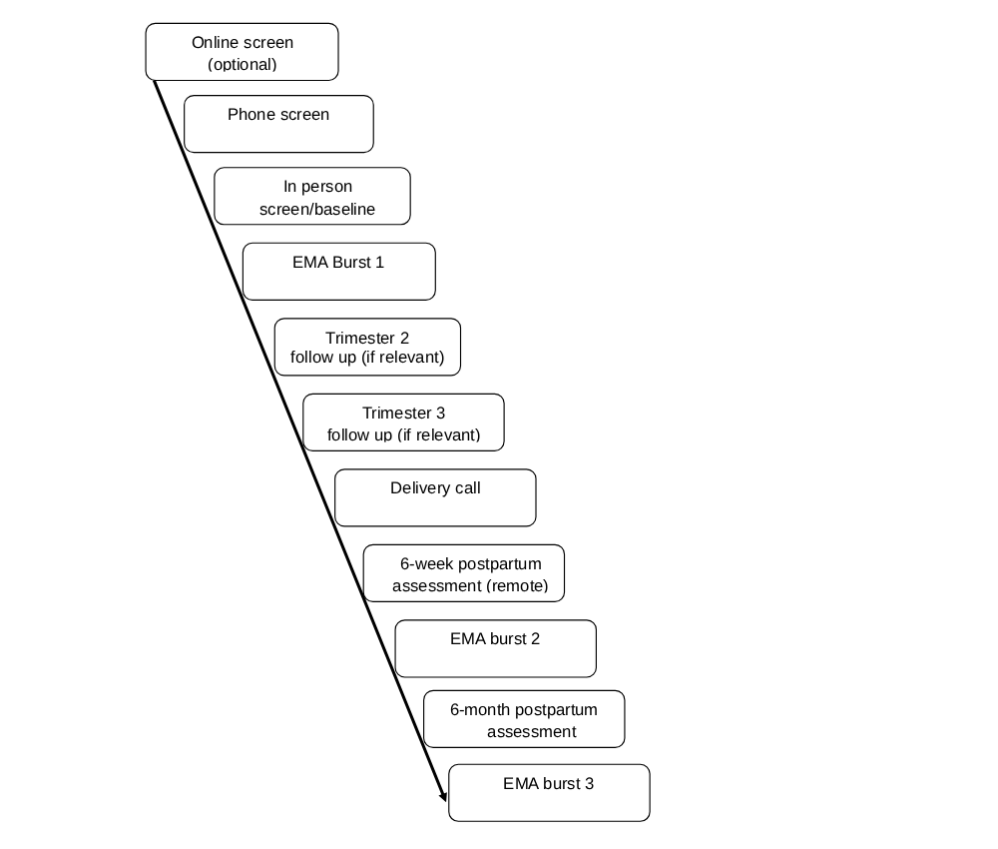
Study flow. EMA: ecological momentary assessment.

Study participation involves a baseline laboratory assessment in pregnancy (before 28-week gestation period; Time 1), a virtual assessment at 6 weeks post partum (Time 2), and a laboratory assessment at 6 months post partum (Time 3). Each Time 1-3 assessment is followed by a 2-week EMA burst for a total of 3 bursts. In addition, up to 2 prenatal retention visits occur in trimesters 2 and 3, such that those enrolled during their first trimester of pregnancy completed follow-ups in both the second and third trimesters, while participants enrolled in their second trimester completed a single follow-up in the third trimester. Participants also completed a brief phone call within 2 weeks post partum, for retention purposes (study flow mentioned in Figure 1).

### Participants

#### Eligibility

Eligibility criteria include (1) participants older than 18 years of age; (2) English-speaking; (3) report cannabis use at least twice weekly over the past 60 days; (4) pregnant with one child with a gestational age no more than 28 weeks (to allow time for scheduling of baseline); (5) not currently in substance use treatment; (6) no current (past month) mania, psychosis, or active suicidal ideation; (7) no substance use other than cannabis, tobacco, or alcohol; and (8) own a personal smartphone to accommodate the data collection app. For revisions to eligibility criteria to address initial recruitment challenges, see Results section.

#### Recruitment and Screening

Participants were recruited through social media, community flyers (posted in locations such as playgrounds, Women, Infants, and Children offices [supplemental nutrition program for women, infants, and children], and obstetrics/gynecology clinics), and via BuildClinical, a platform that identifies potential research participants using online advertising campaigns and conducts initial eligibility screening. Respondents to flyers or online advertisements accessed an online screener hosted on Qualtrics via a clickable link or the QR code. This screener included questions aligned with the study’s inclusion and exclusion criteria. Those meeting initial eligibility criteria were prompted to complete a contact form specifying their preferred method and time for follow-up. Study staff then contacted these individuals to confirm eligibility through a telephone screener. Alternatively, prospective participants could reach out directly via call or text to schedule screening. Eligible participants were invited to an in-person baseline assessment and instructed to abstain from using cannabis for at least 15 hours prior to the visit.

### Procedures

#### In-Person Screening

After verifying identity and age via government-issued ID (eg, driver’s license), participants complete the informed consent process, which was presented via PowerPoint (Microsoft Corp) to facilitate comprehension. After consent is obtained, participants provide biochemical verification of eligibility. Biochemical verification includes a commercially available pregnancy test to confirm pregnancy and a 5-panel iCup urine drug screen which screens for amphetamines, cocaine, methamphetamine, opiates, and tetrahydrocannabinol, as well as validity and specimen integrity checks, including oxidants, pH level, and specific gravity, to confirm cannabis use and no other drug use. Tetrahydrocannabinol is detected at 50 ng/mL. If participants tested negative for tetrahydrocannabinol at 50 ng/mL, a tetrahydrocannabinol dipstick with a greater sensitivity of 5 ng/mL was administered. A saliva test is also conducted to ensure that the participant has not used cannabis in the past 15 hours, with a cut-off threshold of 40 ng/mL of tetrahydrocannabinol. This test was conducted to ensure no intoxication was experienced at study sessions.

Following bioverification of eligibility, the Mini-International Neuropsychiatric Interview (MINI [38]) is administered to rule out past month mania, psychosis, or active suicidal ideation, and the Timeline Followback (TLFB) is administered to rule in twice weekly cannabis use and rule out substance use other than alcohol and nicotine. The MINI is administered by a PhD-level study staff member or a student currently enrolled in an MPH program; both are supervised by a licensed clinical psychologist. Participants deemed ineligible are compensated US $15 and provided psychoeducational resources on cannabis and mental health. These resources are provided to all who were screened and enrolled in the study.

#### Baseline (Time 1) and EMA Burst 1

The baseline assessment lasts approximately 3-4 hours. Participants complete baseline surveys on substance use patterns and psychosocial factors that may influence maternal health. Finally, participants view a PowerPoint presentation and receive hands-on training to familiarize themselves with completing EMA surveys. This training ensured they were comfortable using MetricWire, the EMA mobile app, on their smartphones to report real-time data on daily substance use, mood, and other contextual factors. Participants are subsequently enrolled in the EMA burst procedures, which begin the day following the baseline assessment and last for 2 weeks.

#### Trimester Follow-Ups

Each trimester follow-up assesses recent (30-day) substance use, as well as current depression and anxiety symptoms.

#### Delivery Call

A 5- to 10-minute phone call is conducted 0-14 days post delivery to gather basic information about the newborn. Participants are asked to text research staff upon the delivery of their baby. If staff do not receive a text, they refer to the participants’ estimated delivery date and send text and email reminders to let study staff know when they deliver.

#### 6 Weeks Post Partum (Time 2)

A 30-minute virtual visit occurs at approximately 6 weeks post partum. Participants self-report on substance use, mother-infant bonding, birth experience, infant development, and parental substance use. Participants were refamiliarized with the EMA procedures and are enrolled in EMA burst 2, which begins the day after the Time 2 assessment.

#### 6 Months Post Partum (Time 3)

The participant and their infant attend a 90-minute laboratory assessment at approximately 6 months post partum. The birthing person reports on their substance use, depression, and anxiety. Dyads also complete behavioral tasks, which are video-recorded for later behavioral coding. Following the mother-infant interaction tasks, study staff debrief with the parent to review their experience in the paradigm and ensure mother and baby are comfortable. Participants are refamiliarized with the EMA procedures and are subsequently enrolled in the third and final burst, which begins the day following the 6-month assessment.

#### Ecological Momentary Assessment

EMA data are collected via MetricWire, enabling individualized data collection via participants’ smartphones. At baseline, participants are trained to use the mobile app, including specific instruction on responding to prompts and measurement of cannabis quantity. Participants are instructed to self-initiate reports just before using cannabis (begin cannabis report), which assesses for the formulation of cannabis being used, current reasons for using cannabis, and current mood. After participants complete a begin cannabis report, they are instructed to complete an end use report, and follow-up reports are triggered at 30-minute intervals to assess for the end of the cannabis use event if not self-initiated. Once the end use report is completed, cannabis use event details are assessed, including context, any other substance use, as well as impact on mood and infant presence (postpartum bursts only). Participants also complete random prompts throughout the day (sent randomly within three 3-hour blocks each day) to capture mood during noncannabis use events and cannabis use that is not self-initiated. Participants also complete morning reports, which assess overall sleep quality, mood symptoms, intentions to use cannabis that day, and summarize the prior day’s substance use. Bedtime reports capture mother-child interactions from the day (postpartum bursts only) and any additional use since the last report. Assessments are designed to be less than 2 minutes. Similar methods have been used by Gunn et al [39].

#### Compensation

Participants could earn up to US $595, dispersed on reloadable debit cards. The compensation schedule is outlined in Tables 1 and 2. Payments are provided following each assessment. Compliance is maximized in the EMA study phase by compensating participants by increasing USD amounts based on compliance rates. EMA compliance rates are calculated following the completion of each burst and compliance is compensated following completion of each burst.

**Table 1:** Compensation schedule for non–ecological momentary assessment. Participants deemed ineligible at screening were compensated US $15.

Non-EMA^a^ activities	Compensation amount (US $)
Baseline (in person)	100
Trimester follow-up 1 (if indicated)	25
Trimester follow-up 2 (if indicated)	25
Postdelivery call	10
6-week post partum (virtual)	50
6-month post partum (in person)	85
Study completion bonus^b^	40

^a^EMA: ecological momentary assessment.

^b^Participants receive the completion bonus if they complete all study sessions and at least 50% of surveys across all mobile surveys.

**Table 2:** Compensation schedule for ecological momentary assessment activities.

Percent surveys complete	Burst 1 (US $)	Burst 2 (US $)	Burst 3 (US $)
<25%	10	15	20
25%-49%	30	40	50
50%-74%	60	70	80
75%-89%	70	80	90
90%+	75	85	100

### Measures

A list of constructs assessed and an administration timeline can be found in Tables 3 and 4. Before administering these measures, participants reported their preferred term for cannabis. When applicable, we used the participant’s chosen term in place of “marijuana” throughout the measures.

**Table 3:** Schedule of ecological momentary assessment measures.

EMA^a^ measures	Start	FU^b^	Random	AM^c^	PM^d^
Cannabis form and quantity	✓	✓	✓	✓	✓
Cannabis intoxication		✓	✓		✓
Motives	✓				
Social context		✓	✓		✓
Other substance use		✓			
Mood	✓	✓	✓	✓	✓
Sleep				✓	
Mother-child relationship					✓

^a^EMA: ecological momentary assessment.

^b^FU: follow-up

^c^AM: morning survey.

^d^PM: bedtime survey.

**Table 4:** Schedule of measures.

Construct			Screen		Baseline		Time 2 and Time 3^a^		Delivery		6 Weeks PP^b^		6 Months PP
**Eligibility**													
	Mental health	✓											
	Substance use (past 90-day use)	✓											
	Acute THC^c^ intoxication	✓											
	Urine drug screen	✓											
	Pregnancy	✓											
Demographics					✓						✓		✓
Substance use					✓		✓				✓		✓
Cannabis use beliefs and behaviors					✓								
Mental health					✓		✓				✓		✓
Social support					✓								
Feeding intentions					✓								
Attachment					✓								
Child development									✓		✓		✓
Birth experience											✓		
Parenting stress											✓		✓
Bonding											✓		✓
Mother-infant interaction tasks													✓

^a^Time 2 and Time 3: trimester follow-ups.

^b^PP: post partum.

^c^THC: tetrahydrocannabinol.

### Screening

#### Mental Health: Current Mania, Psychosis, or Suicidal Ideation

The MINI is a short structured diagnostic interview designed to assess major *DSM-IV* (*Diagnostic and Statistical Manual of Mental Disorders* [Fourth Edition]) and *ICD-10* (International Statistical Classification of Diseases and Related Health Problems, Tenth Revision) psychiatric disorders [38]. The MINI assesses for current (past month) mania, psychosis, and suicidal ideation, all of which were exclusion criteria in this study. In addition to these screening data obtained to inform study eligibility, the MINI includes assessments of current and past psychiatric diagnoses, including major depressive disorder, panic disorder, agoraphobia, social anxiety disorder, obsessive compulsive disorder, posttraumatic stress disorder, alcohol and substance use disorder, anorexia nervosa, bulimia nervosa, binge eating disorder, generalized anxiety disorder, and antisocial personality disorder.

#### Substance Use

Participants self-report their substance use with the TLFB, a structured, calendar-based interview tool used to collect detailed retrospective data on substance use [40,41]. Participants are guided to recall daily activities and contextual cues to enhance the accuracy of their reports. The TLFB is included as part of the in-person screening to identify use of cannabis (eligibility), alcohol, tobacco, as well as to capture the use of other substances (ineligibility). Participants also report mode of cannabis use. Duration covered by the TLFB varies by assessment, such that the reporting period was either past 90-day (Time 1) or past 30-day (trimester follow-ups, Time 2, and Time 3) use.

### Baseline

#### Demographics, Household, And Pregnancy Context

Participants report on their demographics (eg, age, biological sex at birth, gender identity, race, ethnicity, income, government assistance), as well as contextual information about the home environment, including the number of children and relationship status. Date of last menstrual period and trimester of pregnancy are queried to determine gestational age. Additional questions probe prenatal care, including the number of prenatal appointments attended and how pregnancy has been confirmed.

#### Other Substance Use

Participants report on their use of nicotine or tobacco products (cigarettes, e-Cigarettes, or nicotine vaporizers, other tobacco or nicotine products) and alcohol use, as well as the frequency and quantity of use for each trimester. Participants report on parallel information about their partner’s substance use (alcohol, cannabis, cigarettes, other tobacco products, and other drugs) since the participant found out they were pregnant.

#### Cannabis Use Characteristics

Participants are asked if they have a medical card, if they use the card to purchase cannabis, and for what conditions they have been provided a medical card. Moreover, they report on their current level of cannabis use (ie, if they use the same, less, or more than when they found out they were pregnant), if they have quit or temporarily stopped using cannabis since finding out they were pregnant, and if so, why.

For each trimester, participants report number of days of cannabis use per week, average times per day of cannabis use, average number of hours high per cannabis use day (presented on a visual analog scale from 0 to 24 hours), how high they felt when using cannabis (visual analog scale ranging from 0=not at all high to 100=as high as possible). Amount of tetrahydrocannabinol versus cannabidiol in cannabis used was assessed (mostly or entirely cannabidiol/no tetrahydrocannabinol, roughly equal parts cannabidiol or tetrahydrocannabinol; mostly or entirely tetrahydrocannabinol or no cannabidiol, or unsure). Finally, participants indicate the number of days (0-7) that they used each of the following: join, hand pipe, bong, blunt, concentrates or dabs, edibles, tetrahydrocannabinol-infused alcoholic beverages, tinctures, and cannabidiol-only products (eg, lotions) [42].

#### Symptom Management Motives

Participants are provided a list of 10 physical symptoms common in pregnancy (ie, stomach or digestion problems, physical pain, neurological symptoms, sleep, fatigue, bleeding, Braxton-Hicks contractions, dyspareunia, hot flashes, and appetite) and 13 emotions (ie, sad or depressed, happy, nervous or anxious, excited, decreased pleasure or motivation, angry, optimistic, lack of energy, fidgety or restless, scared, irritable, and boredom stress). Participants indicated if they did or did not use cannabis for managing each symptom or emotion, and if they reported using cannabis to manage the symptom, they were asked how helpful cannabis was at managing that symptom (response options were did not help, helped somewhat, helped a lot). This measure was developed for this study.

#### Cannabis Consequences

The Brief Marijuana Consequences Questionnaire [43] assesses the negative consequences, grouped into 5 categories: interpersonal, health, academic or occupational, legal, and psychological, associated with cannabis use since the participant found out they are pregnant. Respondents rate how frequently they have experienced each consequence on a 5-point Likert scale (ranging from 0=never to 4=often).

#### Perceived Harms of Substance Use

Participants report on how harmful they think their cannabis use is to their own health, to the health of their baby during pregnancy, and to the health of their baby after they give birth. An additional question probes how harmful they think it is to breastfeed while using cannabis. All questions are presented with separate visual analog scales ranging from 0 (not at all harmful) to 100 (very harmful). In addition, participants are asked the extent to which they agreed or disagreed with statements about the perceived harms of alcohol use, nicotine or tobacco use, and use of other drugs to the developing child. Items were adapted from Nordeng et al [44].

#### Sources of Information and Disclosure About Cannabis Use

Participants are asked where they sought information about substance use and who provided them with information, including unsolicited advice. Response options included physician, midwife, nurse, other health care provider, internet, pregnancy groups, family or friends, dispensaries, or other. A follow-up, open-ended question assessed the type of information received from these sources. Participants also reported if they disclosed their cannabis use to their provider (yes or no), how comfortable they are in discussing their cannabis use with their provider (4-point Likert scale from not at all comfortable to very comfortable), and how honest they have been with their provider about their cannabis use. This measure was developed for this study.

#### Cannabis Stigma

Participants are also asked how much they agree or disagree on a 5-point Likert scale, including a “not sure” option, with the following statements: “there is negative stigma around using cannabis” and “there is negative stigma around using cannabis during pregnancy.” Additionally, participants reported how acceptable or unacceptable they find both medical or therapeutic and recreational cannabis use.

#### Protective Behavioral Strategies

The Protective Behavioral Strategies for Marijuana Scale is a self-report tool designed to assess how often individuals use strategies to minimize potential harms from cannabis use [44,45]. The scale consists of 17 items (eg, “Only use once during a day/night”), each measuring the frequency of specific protective behaviors (response options are never, rarely, occasionally, sometimes, usually, and always). To our knowledge, this is the first application of this measure in a pregnant sample. Together with the developers of the measure, we made 3 modifications to the scale. First, the question stem specifies the use of protective behavioral strategies during pregnancy. Second, replaced “marijuana” with the individual’s preferred term. Finally, we included the following open-ended question to probe for protective behavioral strategies that were not captured in the initial measure: “What other things do you do to help you avoid some of the not so good things about using [preferred cannabis term] while you are pregnant?”

#### Depression

The Edinburgh Postnatal Depression Scale [46] was used to screen symptoms of depression and has been validated in pregnant and postpartum samples [47]. The Edinburgh Postnatal Depression Scale consists of 10 items (eg, “I have felt sad or miserable”) scored on a 4-point Likert scale (0-3), reflecting the frequency or severity of symptoms over the past week. Scores range from 0 to 30, with higher scores indicating greater severity of depressive symptoms.

#### Anxiety

The Perinatal Anxiety Screening Scale (PASS [48]) is designed to screen for a broad range of anxiety symptoms in pregnant and postpartum individuals. The PASS consists of 31 items scored on a 4-point Likert scale ranging from 0 (not at all) to 3 (almost always). The PASS includes four subscales, including excessive worry and specific fears (eg, “Fear that harm will come to the baby”), social anxiety (eg, “Fear that others will judge me negatively”), perfectionism and control (eg, “Needing to be in control of things”), and acute anxiety and trauma. Total scores can range from 0 to 93, with higher scores indicating more severe anxiety symptoms. Anxiety thresholds are defined as low (0-20), moderate (21-41), or high (42+).

#### Social Support

Two questions query social support in pregnancy; the first asked if participants have been adequately supported in their pregnancy (1=never to 5=always), and if they have or would be willing to attend a support group for pregnant individuals. Items were developed for this study.

#### Feeding Intentions

Participants report their intended primary method of feeding their infant during the first 6 months (options: breastfeeding or chestfeeding, pumping, formula-feeding, milk sharing or milk bank, or other). They also complete a check-all-that-apply item listing all feeding methods they planned to use during this period. Participants indicated whether they believe cannabis can pass through breast or chest milk (response options: yes, no, or I don’t know). Items were developed for this study in collaboration with an International Board Certified Lactation Consultant.

#### Attachment

The Maternal-Fetal Attachment Scale is a self-report questionnaire designed to measure the emotional bond that a mother develops with their unborn child during pregnancy [49]. The Maternal-Fetal Attachment Scale assesses the extent to which a pregnant person engages in behaviors that reflect attachment to their fetus. The scale consists of 24 items with four response options (definitely yes, yes, no, and definitely no). The Maternal-Fetal Attachment Scale includes 5 subscales: differentiation of self from fetus, interaction with fetus, attributing characteristics to fetus, giving of self, and role taking. Higher scores indicate stronger maternal-fetal attachment.

### Delivery Call

Newborn information included date of birth, height, weight, Appearance, Pulse, Grimace, Activity, and Respiration scores, head circumference, neonatal intensive care unit admission (yes or no and duration), and sex.

### 6 Weeks Post Partum

#### Demographics and Substance Use Environment

Demographic information collected included the number of children and adults in the home, as well as adult substance use within the household. Participants were presented with a table reflecting the previously reported number of adults in the home and were asked to indicate if any adult had used cannabis, alcohol, cigarettes, other nicotine products, or other drugs since returning home after delivery.

#### Birth Experience

The Childbirth Experience Questionnaire [50] provides a comprehensive assessment of a birthing person’s perceptions and feelings about their labor and birth experience. The measure includes 19 items rated on a 4-point Likert scale ranging from 1 (totally agree) to 4 (totally disagree). The measure consists of 4 key domains: own capacity (eg, I felt strong during labor and childbirth), professional support (eg, my provider understood my needs), perceived safety (eg, I felt scared during labor and birth), and participation (eg, I felt I could have a say in deciding my birthing position).

#### Child Protective Services Involvement

Participants reported on whether or not the family has had Child Protective Services involvement since the birth of the baby.

#### Neonatal Intensive Care Unit

Participants were asked if their baby spent any time in the neonatal intensive care unit, and if so, the number of days.

#### Parenting Stress

The Parental Stress Scale [51] is an 18-item questionnaire assessing parents’ feelings about their parenting role, exploring both positive (eg, emotional benefits and personal development) and negative aspects of parenthood (eg, demands on resources and feelings of stress). Respondents agree or disagree on a 5-point Likert Scale in terms of their typical relationship with their child or children. Example items include “I am happy in my role as a parent” and “I sometimes worry whether I am doing enough for my child(ren).”

#### Infant Temperament

The very short form of the Infant Behavior Questionnaire [52] is a widely used parent-report tool designed to assess temperament in infants aged 3 to 12 months. The measure includes 37 items with response options of 1 (never) to 7 (always), including a not applicable option. Multiple dimensions of temperament are assessed, including activity level, distress to limitations, fear, smiling and laughter, duration of orienting, soothability, and falling reactivity or rate of recovery.

#### Infant Development

Participants completed developmentally appropriate Ages and Stages Questionnaires [53] at 6 weeks and 6 months post partum. This measure is a widely used developmental screening tool designed to assess young children’s developmental progress and identify potential delays or concerns. The 2-month questionnaire (which covers babies ages 1 month 0 days to 2 months 30 days) assesses a child’s development across 5 domains: communication (eg, ability to respond to sounds and voices), gross motor skills (eg, large muscle movements), fine motor skills (eg, hand-eye coordination and small muscle control), problem solving (eg, early cognitive skills), and personal-social skills (eg, social interactions and self-regulatory behaviors). Each domain includes 6 questions that parents answer based on their own observations of their child. Similarly, the 6-month questionnaire assesses a child’s development across the same 5 domains as the 2-month version, but with milestones appropriate for a 6-month-old infant.

#### Bonding

Parents completed 2 questionnaires to identify potential issues in bonding and early signs of difficulties, such as maternal distress or postpartum mood disorders. The Postpartum Bonding Questionnaire [54] includes 25 items that are designed to assess the quality of the emotional bond between a parent, typically the mother, and their newborn. The measure includes 25 items, rated on a 6-point Likert scale from “always” to “never.” Four subscales assess: impaired bonding (eg, “I feel distant from my baby”), rejection and pathological anger (eg, “I feel irritated by my baby”), anxiety about care (eg, “I feel anxious when my baby is with me”), and risk of abuse (eg, “I feel like I might hurt my baby”). The Mother Infant Bonding Scale [55] includes 8 items that reflect aspects of maternal feelings toward the infant (eg, “I feel loving toward my baby”) on a 4-point Likert scale ranging from “Very Much” (0) to “Not at All” (3).

### 6 Months Post Partum

#### Background

Mental health, substance use, parenting stress, bonding, and child development are reassessed at 6 months. Additionally, participants complete 2 behavioral tasks that are video recorded using a 3-camera mounted recording system. This system includes one camera focused on the mother, one on the infant, and one capturing a wide-angle view of the entire room. Video signals from the two cameras are transmitted through a digital mixer to generate a split-screen recording of the mother and infant. The recording process is managed by a research assistant stationed outside the laboratory.

Still-Face Paradigm [56] is a laboratory-based assessment in which a caregiver’s unemotional face is used to evoke pronounced behavioral reactions in the infant. Mothers are seated 18-36 inches from their infants, at eye level with their infants, who are placed in an infant seat by the mother. The paradigm involves three phases of mother-child interaction: (1) Baseline: in the first interaction, mothers are instructed to interact normally with their infants. (2) Still-face: after the mother returns from a break, they are told to keep eye contact but maintain a neutral expression, remain still, and do not touch or interact with their infant. (3) Reunion: after the second break, the mother is instructed to respond to their baby in any way they feel is appropriate. Each episode lasts 120 seconds, and the onset of each episode is cued by a research assistant who is seated in the room, out of sight of the infant.

Mother and infant behaviors are coded using the Infant and Caregiver Engagement Phases framework [57], which includes a set of mutually exclusive phases of interactive engagement and additional regulatory codes based on facial expressions, direction of gaze, and vocalization. The Infant and Caregiver Engagement Phases coding scheme has been applied to the paradigm conducted with substance-exposed infants [57] and infants of mothers experiencing psychiatric symptoms and stress [58]. For infants, engagement codes include passive-withdrawn, protest, object-environment interaction, social monitoring, and social positive engagement. Caregiver engagement codes consist of hostile-intrusive, withdrawn, social monitoring without vocalizing, social monitoring with positive vocalizing, and social positive engagement. Additional infant behaviors coded involve oral self-comforting (eg, mouthing), self-clasping, distancing or turning away, and autonomic stress indicators such as hiccups or spitting up. For caregivers, additional codes capture rough touches and deviations from still-face instructions, including speaking to or touching the infant during the designated still-face period. Infant and mother phases will be coded in two separate viewings of the videotape, with a third viewing dedicated to coding of infant regulatory actions, autonomic stress indicators, mother intrusions, and procedural violations.

#### Free Play

Mothers are instructed to play with a standardized set of developmentally appropriate toys as they normally would at home for 5 minutes. The research assistant is seated outside the room. The free play interaction will be coded using the Emotional Availability Scales [59], based on global judgements from the observers, and designed to assess the emotional connection between parent and child.

### EMA Measures

#### Cannabis Formulation and Quantity

For all assessments, cannabis use is assessed by asking participants whether they used flower, concentrate, or edible (with “select all that apply”), followed by the quantity of cannabis used based on formulation. (ie, flower=grams of flower, concentrate=number of hits, and edible=milligrams of tetrahydrocannabinol). Additionally, participants report the potency of cannabis consumed and the level of intoxication or “high” (“rate how high you feel from 0=not at all high to 10=as high as possible”).

#### Motives for Use

Participants selected all that apply: “to be social,” “to feel less anxious,” “to feel less depressed,” “to relieve physical pain,” “to sleep better,” “to relax,” “to enjoy the effects,” “to increase appetite,” and “other.”

#### Social Context

Participants are prompted to report the context in which they consume cannabis, indicating the number of people they were with, the specific people they were with (eg, friend, partner, and family), and whether or not those individuals were also consuming cannabis at the time.

#### Other Substance Use

Participants are prompted to report any other substance use (ie, “Did you/are you using any other substance?”), reporting these substances via a select all option for alcohol, cigarettes, nicotine vaporizers, other tobacco or nicotine products, simulants, opiates, sedatives, hallucinogens, and other, if applicable.

#### Mood

Participants report on their current mood with an adapted version of the NIH Patient Reported Outcomes Measurement Information System adult depression short [60] form on a 5 point scale: “Rate how much you currently feel this way” from 1=not at all to 5=very much, for depressed, hopelessness, helpless, worthless, anxious, irritable, happy, excited, and relaxed.

#### Mother-Child Relationship

For Time 3 only (ie, post partum), surveys assessed for the following mother-child questions, all reported on a 10 point scale from (not at all to as much as possible): “How bonded did you feel to your child today?” “How emotionally connected did you feel with your baby today?” “How sensitive did you feel toward your baby today?” “When your baby needed something today, how responsive were you to their needs?” “Did you provide the comfort and care that your baby needed today?”

### Data Analysis Plan

Regarding the first objective of this study, to examine the event-level and longitudinal impact of cannabis use on PPD, 2-level linear mixed-effect models (LMEM) will be used to test hypothesis 1, that cannabis use will be associated with reduced negative affect in the moment. Longitudinal regression will be implemented to test hypothesis 2, that greater cannabis use will be associated with greater depression symptoms via the Edinburgh Postnatal Depression Scale across the postnatal period. Cannabis use and PPD symptoms will be modeled as person-level aggregates at each time point. Regarding the second objective of this study, to evaluate the impact of cannabis on mother-infant interactions and depression, LMEM will be used to test hypothesis 3, that cannabis use frequency will be associated with poorer mother-child interaction quality at the day-level, as well as longitudinally using person-level aggregates of cannabis use and laboratory-assessed measures of mother-child interactions. LMEMs will also be used to examine hypothesis 4, that poorer mother-child interaction quality, assessed via the still-face paradigm and free play, will amplify the association between cannabis use and depression, by adding an interaction term to hypothesis 3 models. For all LMEMs, variation in time-varying covariates will be modeled at the event level (level 1). Between-person effects, including aggregates of level 1 predictors and between-person covariates, will be modeled at level 2. Covariates will include EMA compliance (level 2), demographics (level 2), anxiety (level 2), other substance use (level 1), sleep (level 1), birth experience (level 2 postpartum models), and psychotropic medication use (level 2).

### Ethical Considerations

#### Overview

The University institutional review board completed and approved human subjects’ ethics review (approval number 2022003468). Research procedures have been conducted in accordance with the Declaration of Helsinki. All participants provided informed consent, during which a research assistant reviewed all aspects of the study protocol, and knowledge checks were conducted before each participant gave consent. Participants are provided with the opportunity to opt out. Participants have received monetary compensation, outlined below, which was deemed appropriate for the time required to complete study procedures.

#### Relationship to the Parent Research Award

This project is supported by Brown University’s Center for Addiction Disease Risk Exacerbation (CADRE), funded through NIH’s Centers of Biomedical Research Excellence. All projects supported by CADRE benefit from the resources of three cores: the Clinical Laboratory Core, the Recruitment, Engagement, and Community Health Core, and the Administrative Core. The Clinical Laboratory Core provides essential research infrastructure and resources to advance CADRE-funded research. A key objective of this core is to create a database of factors (biological, environmental, social, and behavioral) associated with the development and progression of substance use disorders and chronic disease. In alignment with this objective, the current project incorporated the CADRE Core measures into its data collection process. A complete list of these measures is provided in Multimedia Appendix 1. These measures were administered at the baseline session after the study-specific measures listed below. For the purposes of this report, we focus on the research activities and measures specifically relevant to the aims of the current research.

## Results

This project received institutional review board approval on December 19, 2022, and was awarded funding on February 1, 2023. The recruitment goal of 20 participants was reached on September 4, 2024. Table 5 summarizes the demographic characteristics of the sample. Follow-up data collection is ongoing. At the time of submission, 18 participants completed the full protocol. Retention rates were 100% for trimester follow-ups (20/20), 100% for the delivery call (20/20), and 95% for the 6-month assessment (19/20). One participant was lost to follow-up at the 6-week assessment, indicating a retention rate of 95% (19/20). One participant was pending the final 6-month EMA burst. Data collection is expected to be completed by October 2025, and initial results related to study aims are expected in April 2026. EMA compliance at all 3 bursts was also strong: 80% at burst 1, 81% at burst 2, and 83% at burst 3.

**Table 5:** Participant demographics (N=20).

Demographic variables		Values
Age (years), mean (SD), range		30.15 (5.03), 20-39
**Race**^a^, n (%)		
	White	12 (60)
	African American or Black	2 (10)
	American Indian	1 (5)
	Other^b^	1 (5)
	Multiple	4 (20)
Ethnicity (Latinx), n (%)		6 (30)
**Trimester at recruitment**, n (%)		
	First	4 (20)
	Second	10 (50)
	Third	6 (30)
Cannabis frequency (past 60-day use), mean (SD), range		50.74 (15.87), 14-60

^a^Check all that apply.

^b^One participant (self-identified as Puerto Rican).

**Figure 2:**
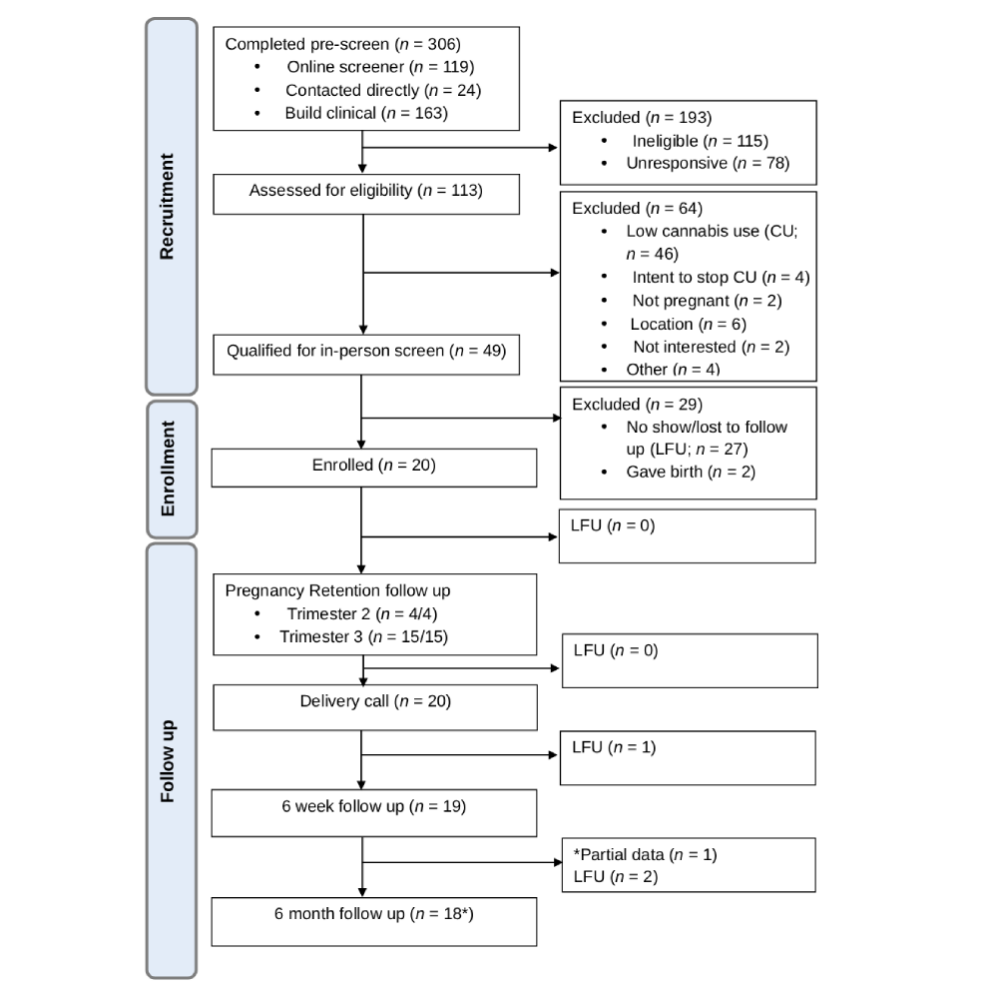
CONSORT (Consolidated Standards of Reporting Trials) flow diagram. CU: cannabis use; LFU: lost to follow up.

A CONSORT (Consolidated Standards of Reporting Trials) flow diagram is presented in Figure 2. We faced significant recruitment challenges upon the initial launch of the project. A total of 3 notable revisions were made to the study procedures and inclusion criteria on April 8, 2024, to alleviate these challenges while retaining the scientific integrity of the project. The first change pertained to the current depression inclusion criteria. At the time of study launch, participants were required to report elevated depression levels, defined as a score of 3 or higher on the Patient Health Questionnaire-2 [61]. We decided to remove this inclusion criterion to allow for the potential capture of depression symptom onset in participants who did not initially screen positive for elevated depression. One participant was recruited prior to changing the depression criterion.

The second change was related to gestational age. Recruitment was initially limited to individuals in their third trimester of pregnancy. This criterion proved challenging because many pregnant individuals we screened reported reducing cannabis use as they approached delivery. The eligibility criterion was expanded to include participants as early as 8 weeks’ gestation. However, this change introduced a significant risk of attrition for those recruited in the first trimester, as their next assessment would not occur until the delivery call. To address this, we incorporated up to 2 retention assessments during the second and third trimesters for participants recruited in the first or second trimester, as applicable. Third, the recruitment platform BuildClinical was implemented in April 2024, which expanded recruitment.

## Discussion

### Overview

This protocol study used a multimethod approach to examine the daily and longitudinal impact of perinatal cannabis use on PPD and the mother-infant relationship. Perinatal cannabis use is increasingly common [62] yet has been linked to heightened risk of adverse maternal mental health outcomes [15]. Similarly, PPD remains one of the most prevalent complications of childbirth, with implications not only for maternal well-being but also for early caregiving and infant socioemotional development [5]. Obtaining repeated measures across the prenatal and postpartum periods allows for a rigorous evaluation of the longitudinal impact of cannabis use on depressive symptoms during a time of heightened vulnerability for maternal mental health.

Preliminary results indicated that retention rates approached 100% and EMA compliance rates exceeded those observed in nonpregnant populations (ie, >80% across all EMA bursts). These high rates, suggestive of strong feasibility and acceptability, demonstrate the ability to successfully implement a rigorous study procedure that captures both momentary and cumulative associations between cannabis use and PPD, as well as downstream effects on the mother-infant relationship. Pending analyses resulting from these data are thus well-positioned to provide preliminary evidence on how cannabis use may shape depressive symptoms in-the-moment and over time across the postpartum period.

### Study Implications

This study advances current research by introducing several key innovations that enhance our understanding of the relationship between cannabis use, PPD, and mother-child bonding or interactions. First, the study uses an ecologically valid methodology by using EMA to investigate the impact of cannabis use on PPD and mother-child interactions within real-world environments. Traditional between-person designs are not suited to capturing dynamic, within-person effects across varying contexts and over time, as is possible here. By adopting EMA methods, this study addresses calls for research that explores caregiver-child interactions in natural settings, offering a more authentic and nuanced perspective [37]. Further, we identified compliance rates higher than standard substance use EMA studies [63], suggesting excellent feasibility of these methods in perinatal populations. Second, this study is the first to explore the impact of cannabis use on key markers of quality of the mother-infant relationship and how these may contribute to maternal depression in a potentially cyclical manner. Understanding this directional association will provide essential preliminary data to inform future longitudinal research examining these dynamics over extended periods of child development. Finally, the study integrates a multimethod assessment approach by combining ambulatory assessment with laboratory and self-reported measures to evaluate mother-child interactions comprehensively. This approach captures the complexity of parenting behaviors in real-world contexts, which has not been achieved in previous studies. Together, these innovations will generate critical insights into how cannabis use and PPD influence mother-child interactions, laying the groundwork for future research and potential interventions.

### Limitations

This study has limitations that should be considered. First, the inclusion of participants who use other substances, such as alcohol and tobacco, may complicate the parsing of individual substance effects. However, since the study does not focus on substance-specific outcomes (eg, metabolism), this approach aligns with the reality of perinatal polysubstance use and improves generalizability. Second, the lack of a comparison group is a limitation driven by the scope of the current project. Inclusion of a control group, which is the next step for this work, would allow for direct comparisons between mothers who use cannabis and those who do not, providing a clearer understanding of the specific impact of cannabis use on PPD and the mother-child relationship. This comparison would help isolate the effects of cannabis from other confounding variables, such as general perinatal stress. Third, the study does not assess parenting behaviors while mothers are acutely under the influence of cannabis. While this research provides valuable insights into the momentary relationship between cannabis use, PPD, and eventual mother-child interactions, it does not directly examine the immediate, short-term effects of cannabis use on maternal behavior and caregiving in real-time. For instance, cannabis may affect emotional regulation, attentiveness, and responsiveness during critical caregiving moments, which could have important implications for child development and maternal mental health. Future research could address this gap by investigating how acute cannabis use influences maternal behaviors and interactions with the infant, in real-time, as well as the potential for short-term disruptions in mother-child bonding or engagement during cannabis intoxication. Fourth, the large battery of assessments related to the parent study may limit generalizability to samples for whom this level of observation may represent a significant burden. Future studies expanding this work should consider the population of interest fully when determining study assessments. Finally, we opted not to exclude participants using prescribed antidepressants. The inclusion of participants using antidepressants may make it challenging to disentangle the effects of medication from those of cannabis use on PPD. However, excluding these participants would significantly limit the generalizability of the findings to real-world populations in which antidepressant use is common.

### Conclusions

This study offers a significant step forward in understanding the relationship between cannabis use, PPD, and critical aspects of the mother-child relationship in real-world contexts. By using naturalistic and laboratory methods, the study captures both the dynamic and nuanced nature of maternal behavior and mood in natural environments, as well as in tightly controlled settings. Ultimately, this work lays the groundwork for developing targeted interventions to support maternal well-being and healthy early relationships between mothers and their children.

## Appendix

Multimedia Appendix 1 CADRE Core measures administered in this project.

## Abbreviations

CADRE: Center for Addiction Disease Risk Exacerbation
CONSORT: Consolidated Standards of Reporting Trials
DSM-IV: Diagnostic and Statistical Manual of Mental Disorders (Fourth Edition)
EMA: ecological momentary assessment
ICD-10: International Statistical Classification of Diseases and Related Health Problems, Tenth Revision
LMEM: linear mixed-effect model
MINI: Mini-International Neuropsychiatric Interview
PASS: Perinatal Anxiety Screening Scale
PPD: postpartum depression
TLFB: Timeline Followback

## Data Availability

Data will be deposited in a publicly available repository.
