# Supplementary material for: Perinatal Cannabis Use, Depression, and the Mother-Child Dyad: Protocol for a Prospective Multimethod Study
Source: JMIR Res Protoc. 2025 Dec 11;14:e71302. doi: 10.2196/71302 (PMC12741660; doi:10.2196/71302)
Supplement: Multimedia Appendix 1 [file resprot_v14i1e71302_app1.docx]

**Table S1.**

| **Measure** | **Citation/Details** |
| --- | --- |
| **Physiological Measures** | |
| Inflammation | Ella Automated Immunoassay System *(ProteinSimple, San Jose, CA)* |
| *interleukin-6 (IL-6)* | Ella Automated Immunoassay System *(ProteinSimple, San Jose, CA)* |
| *interleukin-10 (IL-10)* | Ella Automated Immunoassay System *(ProteinSimple, San Jose, CA)* |
| *interleukin-17A (IL-17A)* | Ella Automated Immunoassay System *(ProteinSimple, San Jose, CA)* |
| *tumor necrosis factor-alpha (TNF-α)* | Ella Automated Immunoassay System *(ProteinSimple, San Jose, CA)* |
| *Interferon-gamma (INFɣ)* | Ella Automated Immunoassay System *(ProteinSimple, San Jose, CA)* |
| *Monocyte chemoattractant protein 1(MCP-1/CCL2)* | Ella Automated Immunoassay System *(ProteinSimple, San Jose, CA)* |
| *Brain-Derived Neurotrophic Factor (BDNF)* | Ella Automated Immunoassay System *(ProteinSimple, San Jose, CA)* |
| Breath carbon monoxide (CO) | *Smokerlyzer ED50*  *(Bedfont Scientific, Haddonfield, NJ)* |
| **Administered in Interview Format** | |
| **Questionnaires** | **References** |
| Adverse Childhood Experience Questionnaire | [1] |
| Brief Medical History Questionnaire | N/A |
| Brief Trauma Questionnaire | [2] |
| HIV Risk-Taking Behavior Scale | [3] |
| Mini International Neuropsychiatric Interview for DSM-5 (MINI 7.0.2) | [4,5] |
| Timeline Followback | [6] |
| **Self-Administered** | |
| American Thoracic Society Questionnaire | [7] |
| Behavioral Risk Factor Surveillance System  (BRFSS) Questionnaire | [8] |
| Demographics | N/A |
| Everyday Discrimination Scale (short version) | [9,10] |
| Fagerström Test of Cigarette Dependence | [11] |
| Family Tree Questionnaire | [12] |
| General Anxiety Disorder-7 (GAD-7) | [13] |
| Godin Exercise Questionnaire | [14] |
| Major Discrimination Questionnaire | [9] |
| Monetary Choice Questionnaire | [15] |
| Pain Catastrophizing Scale | [16] |
| Patient Health Questionnaire (PHQ-9) | [17] |
| Penn State Electronic Cigarette Dependence Index | [18] |
| Perceived Stress Scale | [19] |
| Pittsburgh Sleep Quality Index | [20] |
| PROMIS(r) Pain Intensity Scale | [21] |
| PROMIS(r) Pain Interference Scale | [21] |
| Rapid Eating & Activity Assessment | [22] |
| Reward Probability Index | [23] |
| Short Form Health Survey | [24] |
| Snaith-Hamilton Pleasure Scale | [25] |
| Time Perspectives Questionnaire | [26] |
| Tobacco Use History and Exposure | [27] |

*Note: Additional physiological measures (e.g., blood pressure) are assessed in other CADRE-funded projects, but were not assessed here due to the nature of the sample.*

### **References**

1. Felitti, V. J., Anda, R. F., Nordenberg, D., Williamson, D. F., Spitz, A. M., Edwards, V., Koss, M. P., & Marks, J. S. (1998). Relationship of childhood abuse and household dysfunction to many of the leading causes of death in adults. The Adverse Childhood Experiences (ACE) Study. *American journal of preventive medicine*, *14*(4), 245–258. <https://doi.org/10.1016/s0749-3797(98)00017-8>
2. Schnurr, P. P., Spiro, A. III, Vielhauer, M. J., Findler, M. N., & Hamblen, J. L. (2002). Trauma in the lives of older men: Findings from the Normative Aging Study. *Journal of Clinical Geropsychology, 8*(3), 175–187. <https://doi.org/10.1023/A:1015992110544>
3. Darke, S., Hall, W., Heather, N., Ward, J., & Wodak, A. (1991). The reliability and validity of a scale to measure HIV risk-taking behaviour among intravenous drug users. *AIDS (London, England)*, *5*(2), 181–185. <https://doi.org/10.1097/00002030-199102000-00008>
4. Sheehan D V, Lecrubier Y, Sheehan K H, Amorim P, Janavs J, Weiller E, Hergueta T, Baker R, Dunbar G C. The mini-international neuropsychiatric interview (M.I.N.I.): the development and validation of a structured diagnostic psychiatric interview for DSM-IV and ICD-10. J Clin Psychiatry 1998; 59 Suppl 20:22-33;quiz 34
5. Robinson, S. M., Sobell, L. C., Sobell, M. B., & Leo, G. I. (2014). Reliability of the Timeline Followback for cocaine, cannabis, and cigarette use. *Psychology of Addictive Behaviors, 28*(1), 154–162. <https://doi.org/10.1037/a0030992>
6. Sobell, L. C., Sobell, M. B., Leo, G. I., & Cancilla, A. (1988). Reliability of a timeline method: assessing normal drinkers' reports of recent drinking and a comparative evaluation across several populations. *British Journal of Addiction, 83*(4), 393–402. <https://doi.org/10.1111/j.1360-0443.1988.tb00485>.
7. Comstock, G. W., Tockman, M. S., Helsing, K. J., & Hennesy, K. M. (1979). Standardized respiratory questionnaires: comparison of the old with the new. *The American review of respiratory disease*, *119*(1), 45–53. <https://doi.org/10.1164/arrd.1979.119.1.45>
8. Centers for Disease Control and Prevention (2017). *Behavioral Risk Factor Surveillance System Survey Questionnaire*. Atlanta, Georgia: U.S. Department of Health and Human Services, Centers for Disease Control and Prevention.
9. Sternthal, M. J., Slopen, N., & Williams, D. R. (2011). Racial disparities in health: How much does stress really matter? *Du Bois review: Social Science Research on Race*, *8*(1), 95–113. <https://doi.org/10.1017/S1742058X11000087>
10. Fagerström K. (2012). Determinants of tobacco use and renaming the FTND to the Fagerstrom Test for Cigarette Dependence. *Nicotine & Tobacco Research, 14*(1), 75–78. <https://doi.org/10.1093/ntr/ntr137>
11. Heatherton, T. F., Kozlowski, L. T., Frecker, R. C., & Fagerström, K. O. (1991). The Fagerström Test for Nicotine Dependence: a revision of the Fagerström Tolerance Questionnaire. *British journal of addiction*, *86*(9), 1119–1127. <https://doi.org/10.1111/j.1360-0443.1991.tb01879>.
12. Mann, R. E., Sobell, L. C., Sobell, M. B., & Pavan, D. (1985). Reliability of a family tree questionnaire for assessing family history of alcohol problems. *Drug and Alcohol Dependence*, *15*(1-2), 61–67. <https://doi.org/10.1016/0376-8716(85)90030-4>
13. Spitzer, R. L., Kroenke, K., Williams, J. B., & Löwe, B. (2006). A brief measure for assessing generalized anxiety disorder: the GAD-7. *Archives of Internal Medicine*, *166*(10), 1092–1097. <https://doi.org/10.1001/archinte.166.10.1092>
14. Godin, G., & Shephard, R. J. (1985). A simple method to assess exercise behavior in the community. *Canadian journal of applied sport sciences, 10*(3), 141–146.
15. Kirby, K. N., Petry, N. M., & Bickel, W. K. (1999). Heroin addicts have higher discount rates for delayed rewards than non-drug-using controls. *Journal of Experimental Psychology. General*, *128*(1), 78–87. <https://doi.org/10.1037//0096-3445.128.1.78>
16. Sullivan MJL, Bishop S, Pivik J. (1995). The Pain Catastrophizing Scale: Development and validation. *Psychol Assess, 7,* 432–524.
17. Kroenke, K., Spitzer, R. L., & Williams, J. B. (2001). The PHQ-9: validity of a brief depression severity measure. *Journal of General Internal Medicine*, *16*(9), 606–613. <https://doi.org/10.1046/j.1525-1497.2001.016009606>.
18. Foulds, J., Veldheer, S., Yingst, J., Hrabovsky, S., Wilson, S. J., Nichols, T. T., & Eissenberg, T. (2015). Development of a questionnaire for assessing dependence on electronic cigarettes among a large sample of ex-smoking E-cigarette users. *Nicotine & Tobacco Research, 17*(2), 186–192. <https://doi.org/10.1093/ntr/ntu204>
19. Cohen, S., Kamarck, T., & Mermelstein, R. (1983). A global measure of perceived stress. *Journal of Health and Social Behavior*, *24*(4), 385–396.
20. Buysse, D. J., Reynolds, C. F., 3rd, Monk, T. H., Berman, S. R., & Kupfer, D. J. (1989). The Pittsburgh Sleep Quality Index: a new instrument for psychiatric practice and research. *Psychiatry research*, *28*(2), 193–213. <https://doi.org/10.1016/0165-1781(89)90047-4>
21. Dworkin, R. H., Turk, D. C., Farrar, J. T., Haythornthwaite, J. A., Jensen, M. P., Katz, N. P., Kerns, R. D., Stucki, G., Allen, R. R., Bellamy, N., Carr, D. B., Chandler, J., Cowan, P., Dionne, R., Galer, B. S., Hertz, S., Jadad, A. R., Kramer, L. D., Manning, D. C., Martin, S., … IMMPACT (2005). Core outcome measures for chronic pain clinical trials: IMMPACT recommendations. *Pain*, *113*(1-2), 9–19. <https://doi.org/10.1016/j.pain.2004.09.012>
22. Gans, K. M., Risica, P. M., Wylie-Rosett, J., Ross, E. M., Strolla, L. O., McMurray, J., & Eaton, C. B. (2006). Development and evaluation of the nutrition component of the Rapid Eating and Activity Assessment for Patients (REAP): A new tool for primary care providers. *Journal of Nutrition Education and Behavior, 38*(5), 286–292. <https://doi.org/10.1016/j.jneb.2005.12.002>
23. Carvalho, J. P., Gawrysiak, M. J., Hellmuth, J. C., McNulty, J. K., Magidson, J. F., Lejuez, C. W., & Hopko, D. R. (2011). The reward probability index: Design and validation of a scale measuring access to environmental reward. *Behavior Therapy*, *42*(2), 249–262. <https://doi.org/10.1016/j.beth.2010.05.004>
24. Ware, J. E., Jr, & Sherbourne, C. D. (1992). The MOS 36-item short-form health survey (SF-36). I. Conceptual framework and item selection. *Medical Care*, *30*(6), 473–483.
25. Snaith, R. P., Hamilton, M., Morley, S., Humayan, A., Hargreaves, D., & Trigwell, P. (1995). A scale for the assessment of hedonic tone the Snaith-Hamilton Pleasure Scale. *The British Journal of Psychiatry, 167*(1), 99–103. <https://doi.org/10.1192/bjp.167.1.99>
26. Hall, P. A., Fong, G. T., & Sansone, G. (2015). Time perspective as a predictor of healthy behaviors and disease-mediating states. In M. Stolarski, N. Fieulaine, & W. van Beek (Eds.), Time perspective theory; Review, research and application: Essays in honor of Philip G. Zimbardo (pp. 339–352). Springer International Publishing/Springer Nature. [https://doi.org/10.1007/978-3-319-07368-2_22](https://psycnet.apa.org/doi/10.1007/978-3-319-07368-2_22)
27. Stolarski, N. Fieulaine, & W. van Beek (Eds.), Time perspective theory; Review, research and application: Essays in honor of Philip G. Zimbardo (pp. 339–352). Springer International Publishing/Springer Nature. [https://doi.org/10.1007/978-3-319-07368-2_22](https://psycnet.apa.org/doi/10.1007/978-3-319-07368-2_22)
